# Febuxostat Versus Allopurinol Use in Gout Patients: A Real-World Study

**DOI:** 10.7759/cureus.95499

**Published:** 2025-10-27

**Authors:** Josephin Voskamp, Anne-Kathrin Tausche

**Affiliations:** 1 Internal Medicine/Rheumatology, Dresden University Hospital, Dresden, DEU

**Keywords:** allopurinol, cardiovascular events, febuxostat, gout, uric acid

## Abstract

Introduction/objectives: The primary goal of this study is the epidemiological observation and characterisation of a gout patient cohort. Following this, allopurinol, febuxostat, or no uric acid-lowering treatment was given, and cardiovascular events and deaths during the follow-up were noted. We precisely documented and studied each patient’s comorbidities and co-medications for the analysis.

Method: Our analysis included 208 gout patients, focusing on their epidemiological and clinical features. We monitored the treatment progress of 155 patients using the prescribed medications. Patients received treatment in the rheumatology department at Dresden University Hospital. On average, participants were followed up for 248.7 weeks (4.8 years). We conducted an association study, grouping participants based on their primary uric acid-lowering medication (if they used it for at least 60% of the follow-up period).

Results: In the follow-up, 7.5% of patients in the allopurinol group (five patients) and 18.1% (13 patients) in the febuxostat group experienced at least one cardiovascular event. Among participants primarily using allopurinol, four deaths (6%) were recorded, compared to five deaths (6.9%) among those mainly using febuxostat.

Conclusions: While the febuxostat group showed more events, this was associated with a higher prevalence of almost all risk factors. Interpreting study results and applying them practically remain difficult given the constellation’s commonality in clinical life; therefore, careful consideration is necessary.

## Introduction

Given our typical meat-heavy Western diet, the resurgence of gout, a painful joint condition caused by urate crystal buildup, is not surprising. Every year, more patients are hospitalised with gout than with rheumatoid arthritis [1]. Gout is classified as part of the metabolic syndrome and is associated with cardiovascular diseases. Because of the constant subclinical inflammation, it is also an independent risk factor for the latter [2,3]. The cardiovascular risk of the patients is 50 to 70% higher, with an increased mortality compared to the general population [4]. The pre-existing condition of gout raises the risk of atherosclerosis [2], deep vein thrombosis and pulmonary embolism [4].

With optimal treatment, we can prevent chronic arthritis and uric acid nephropathy and eliminate the lowering of life expectancy caused by gout [5], given that well-standardised and effective therapeutic options are established [2]. Sadly, health service research shows that suboptimal management of gout still exists; there are various reasons for this [1,2]. Often, a uric acid-lowering treatment is not initiated even though the sign is given, or no further checkups of the serum uric acid level occur. The current first-line treatment comprises allopurinol, a xanthine oxidase Inhibitor. It is one of the most prescribed drugs and is well tolerated [6]. Studies showed that allopurinol lowers the cardiovascular risk of gout patients [7-9]. A well-known rare side effect is the allopurinol hypersensitivity syndrome [6], which presents with dermal symptoms and the possibility of an associated multiple organ failure with a mortality rate of 14%. It is also the most common cause of the life-threatening Stevens-Johnson syndrome [3]. A much more common problem is the fact that in a significant fraction of patients taking allopurinol, the targeted concentration of the serum uric acid is not achieved, so that crystals do not dissolve, which may either result from poor adherence to therapy or the quite common (physician-directed) underdosing of the drug [3].

Febuxostat, which is the subject of current debates and this paper, is also a xanthine oxidase Inhibitor and has been available since 2008. It presented promising results in its pivotal trials and showed a more effective uric acid-lowering profile than the commonly used dosage of allopurinol [10]. The German guideline suggests a prescription with intolerance of allopurinol, if the targeted uric acid levels cannot be reached using allopurinol or if the patient suffers from impaired renal function [3]. Since febuxostat is mostly metabolised through the liver, there is no need to lower the dosage in the presence of renal impairment [2]. Cases of hypersensitivity and drug interactions are less common than in patients taking allopurinol [3,11]. Because there was a (non-significant) higher number of deaths in the febuxostat group than in the allopurinol group in the phase-III-pivotal trial [12], the US Food and Drug Administration (FDA) demanded a study to determine the cardiovascular safety of febuxostat [13]. Although the CARES trial (2018) showed no significant difference in primary endpoints, febuxostat’s performance was significantly worse than that of allopurinol in terms of cardiovascular mortality. The consequences of this study were controversially discussed, since there was a high rate of loss to follow-up and the events mostly occurred in the first thirty days after discontinuation of the study drug [14]. The European Medicines Agency (EMA) also demanded a safety study: the FAST trial showed, in contrast to CARES, no differences in the cardiovascular safety of allopurinol and febuxostat; however, only 14% of the included patients suffered from a cardiovascular comorbidity [15]. Currently, we can find limitations to the usage of febuxostat in gout patients with underlying cardiovascular diseases both in the prescribing information and the official approval of the drug [10].

So now, we have to answer the question of how the findings of trials like this can be used in clinical routines and how doctors should implement that information concerning the often-complex requirements of individual patients, also considering the treat-to-target strategy recommended by current guidelines [4].

## Materials and methods

We initially included 208 patients with a confirmed gout diagnosis secured by polarising light microscopy from the Euro-Gout cohort, which we first anonymised and then described, analysed and interpreted considering their epidemiological and clinical characteristics. In the second part of this study, we examined the further progression of these patients regarding their treatment with allopurinol, febuxostat, or with no uric acid-lowering therapy. For this part, we could only include 155 of the original participants: 46 patients were not available to follow up, and six had to be excluded because of non-compliance. The average period of observation for this was 248.7 weeks (4.8 years), with a maximum of 412 weeks (7.9 years) and a minimum of 19 weeks (0.4 years). The participants were all seen by gout specialists of the rheumatology department at Dresden University Hospital; thus, our cohort comprises patients with a more severe or complicated gout disease than the average patient who is normally treated by their primary care physician. To gather the information for the follow-up, we developed a survey, which included the development of the disease and the uric acid-lowering treatment over the years and various cardiovascular data, e.g., blood pressure, the occurrence of major adverse cardiovascular events (MACEs) or the intake of certain medications. MACE was defined as myocardial infarction, cardiac or cerebral revascularisation procedure, cardiac arrhythmias, stroke, transient ischemic attack or peripheral vascular disease. These were then collected through Orbis (the patient management system of the university hospital), captured in Microsoft Excel (Microsoft Corporation, Redmond, USA) and analysed using SAS. As is common under real-life circumstances, our patients did not receive the same drug over several years in the same dosage but underwent various changes in both dosage and, as the circumstances required, also the type of uric acid-lowering substance. Considering this, we could not perform the usual evaluation methods as per intention-to-treat or per-protocol analysis. Instead, we performed an association study and classified the patients into groups according to their dominant uric acid-lowering medication (≥60% of the follow-up). We also had to form a group of participants who, most of the time, did not take any gout treatment and one for those who, because of various changes in their medication, could not be included in one of the other groups. This method left us with the following numbers: 67 patients (43.2%) taking mostly allopurinol, 72 patients (46.5%) taking mostly febuxostat, seven patients (4.5%) taking neither of those most of the time and nine patients (5.8%) with an often changing type of drug.

## Results

The characteristics of the patients at the start of the study, categorised by sex, are seen in Table 1. The mean age of our patients at the time of the screening was 59.3 years, 87.5% (n=182) were male and 12.5% (n=26) were female. They suffered from their first gout attack at a median age of 44.4 years, and the previous duration of the disease averaged 14.8 years. 46.1% (n=96) of the participants stated a known history of gout in their family, and 94.7% (n=197) a monoarticular manifestation (as in there was only one joint affected during an attack). Regarding their comorbidities, 23.1% (n=48) of the patients suffered from impaired renal function, 24% (n=50) from diabetes mellitus type II, 77% (n=160) from arterial hypertension, 54.5% (n=113) from dyslipidemia, 23.4% (n=49) from coronary heart disease and 3.4% (n=7) from heart failure. A previous stroke happened to 5% (n=10) and a previous myocardial infarction to 8.4% (n=17) of the participants. Overall, 87% (n=181) presented with at least one of the mentioned comorbidities. Corresponding to this profile, 66.4% (n=138) of the patients were taking at the minimum one antihypertensive drug, 38% (n=79) at least one lipid-lowering agent, 15.4% (n=32) at least one antidiabetic, 9.6% (n=20) at least one diuretic and 22.6% (n=47) were prescribed low-dose aspirin. Only 27.4% (n=57) claimed to have no co-medication during the first visit. The mean eGFR was 74.9ml/min, with a minimum of 9.4ml/min and a maximum of 124ml/min. Meanwhile, the average serum uric acid levels were measured at 380µmol/l with a range from 100µmol/l to 880µmol/l.

**Table 1:** Characteristics of the patients at the start of the study, categorised by sex eGFR: Estimated glomerular filtration rate

	Female patients (n=26)	Male patients (n=182)	All patients (n=208)
Percentage	12.5%	87.5%	100%
Age	66.6 years	58.2 years	59.3 years
Age during the initial gout attack	51.2 years	43.4 years	44.4 years
Previous duration of gout	16.3 years	14.5 years	14.8 years
Family history of gout	42.3% (n=11)	45.6% (n=83)	46.1% (n=96)
Polyarticular gout manifestation	3.8% (n=1)	5.6% (n=10)	5.3% (n=11)
Impaired renal function	50% (n=13)	19.2% (n=35)	23.1% (n=48)
Diabetes mellitus type II	38.5% (n=10)	22% (n=40)	24% (n=50)
Arterial hypertension	84.6% (n=22)	75.8% (n=138)	77% (n=160)
Dyslipidemia	57.7% (n=15)	52.2% (n=95)	54.5% (n=113)
Coronary heart disease	26.9% (n=7)	22.5% (n=41)	23.4% (n=49)
Heart failure	3.8% (n=1)	3.3% (n=6)	3.4% (n=7)
Previous stroke	0%	5.5% (n=10)	5% (n=10)
Previous myocardial infarction	7.7% (n=2)	8.2% (n=15)	8.4% (n=17)
At least one antidiabetic drug	26.9% (n=7)	13.7% (n=25)	15.4% (n=32)
At least one antihypertensive drug	73.1% (n=19)	65.4% (n=119)	66.4% (n=138)
At least one lipid-lowering agent	46.2% (n=12)	36.8% (n=67)	38% (n=79)
At least one diuretic	19.2% (n=5)	8.2% (n=15)	9.6% (n=20)
eGFR	55.2ml/min	77.6ml/min	74.9ml/min
Serum uric acid level (mean)	380µmol/l	380µmol/l	380µmol/l

As already mentioned, we could not follow up on all the 208 patients that we analysed for the first part; that's why the number of participants came down to 155. The groups of the analyses of the second part were conducted according to the uric acid-lowering treatment taken at least 60% of the observed time, which added up to the following numbers: allopurinol group: 67 patients (43.2%); febuxostat group: 72 patients (46.5%); no uric acid-lowering agents: seven patients (4.5%); and a group with a balanced ratio of taking allopurinol, febuxostat or neither of those: nine patients (5.8%).

We found that the patients taking febuxostat had a higher rate of diabetes (31.9% (n=23) vs. 22.4% (n=15)) and atrial fibrillation (26.4% (n=19) vs. 17.9% (n=12)) than the ones taking allopurinol, while the lowest rate of both could be observed in the participants taking no uric acid-lowering medication (Table 2). The febuxostat group also had the highest percentage of antihypertensive drugs (90.3% (n=65); allopurinol: 74.6% (n=50); no medication: 28.6% (n=2)), diuretics (febuxostat: 43.1% (n=31); allopurinol: 35.8% (n=24); no medication: 14.3% (n=1)), anticoagulants (febuxostat: 52.8% (n=38); allopurinol: 35.8% (n=24); no medication: 42.9% (n=3)) and lipid-lowering agents (febuxostat: 43.1% (n=31); allopurinol: 38.8% (n=26); no medication: 42.9% (n=3)). There was a higher rate of patients with no uric acid-lowering drugs taking at least one antidiabetic (28.9% (n=2)) but the febuxostat group still ended up with a greater percentage than the allopurinol group (11.1% (n=8) vs. 9% (n=6)).

**Table 2:** Comorbidities and co-medications

	Allopurinol	Febuxostat	No uric acid-lowering drugs	Balanced drug application
Total rate n (%)	67 (43.2%)	72 (46.5%)	7 (4.5%)	9 (5.8%)
Diabetes mellitus type II	22.4% (n=15)	31.9% (n=23)	14.3% (n=1)	55.6% (n=5)
Atrial fibrillation	17.9% (n=12)	26.4% (n=19)	0%	22.2% (n=2)
At least one antihypertensive drug	74.6% (n=50)	90.3% (n=65)	28.6% (n=2)	77.8% (n=7)
At least one diuretic	35.8% (n=24)	43.1% (n=31)	14.3% (n=1)	44.4% (n=4)
At least one anticoagulant	35.8% (n=24)	52.8% (n=38)	42.9% (n=3)	44.4% (n=4)
At least one lipid-lowering agent	38.8% (n=26)	43.1% (n=31)	42.9% (n=3)	44.4% (n=4)
At least one antidiabetic drug	9% (n=6)	11.1% (n=8)	28.9% (n=2)	11.1% (n=1)

Elevated liver enzymes during the therapy were documented in 37.3% (n=25) of the patients taking mostly allopurinol and 27.8% (n=20) of the ones taking mostly febuxostat (Table 3). There was an even higher percentage in the group with no uric acid-lowering medication (42.9% (n=3)) and the balanced group (44.4% (n=4)). Only two participants (2.8%) in the febuxostat group displayed side effects that could directly be associated with the uric acid-lowering therapy. One patient complained about a dry mouth and one about muscle cramps, both of which disappeared after lowering the dosage of the drug. 13 patients (18.1%) taking mostly febuxostat, and five patients (7.5%) taking allopurinol over 60% of the time reported at least one MACE. There were five deaths (6.9%) in the febuxostat group and four deaths (6%) in the allopurinol group, and one of each group was assigned to a cardiovascular cause. We documented one death (11.1%) in the balanced group and none among the patients who took no uric acid-lowering medication most of the time.

**Table 3:** Major adverse cardiovascular events (MACEs), side effects, and deaths

	Allopurinol	Febuxostat	No uric acid-lowering drugs	Balanced drug application
Total rate n (%)	67 (43.2%)	72 (46.5%)	7 (4.5%)	9 (5.8%)
Elevated liver enzymes	37.3% (n=25)	27.8% (n=20)	42.9% (n=3)	44.4% (n=4)
Side effects	0%	2,8% (n=2)	0%	0%
At least one MACE	7.5% (n=5)	18.1% (n=13)	14.3% (n=1)	33.3% (n=3)
Death	6% (n=4)	6.9% (n=5)	0% (n=0)	11.1% (n=1)

Through our results, we could see a notable correlation between the number of prescribed antihypertensive and antidiabetic drugs, anticoagulants and lipid-lowering agents and the occurrence of MACEs (Table 4). A similar effect can be noted up to two different diuretics. It is worth mentioning that there were only three patients included in our analyses who were taking three or four diuretics. We want to emphasise that the highest rates of diuretics, anticoagulants, lipid-lowering agents, antihypertensive drugs and antidiabetic drugs were found in the febuxostat group.

**Table 4:** Rates of MACEs and death according to the number of different co-medications taken MACE: Major adverse cardiovascular event

Number of co-medications	0	1	2	3	4
Antihypertensive drugs	0%	10.6% (n=5)	15.6% (n=7)	25% (n=6)	50% (n=4)
Diuretics	10.5% (n=10)	16.7% (n=7)	33.3% (n=5)	0%	0%
Anticoagulants	2.3% (n=2)	21.7% (n=13)	75% (n=6)	100% (n=1)	
Lipid-lowering agents	6.6% (n=6)	23.7% (n=14)	40% (n=2)		
Antidiabetic drugs	13% (n=18)	25% (n=4)	0%		

There also was a notable association regarding co-morbid renal diseases, diabetes, coronary heart disease, heart failure, and a previous stroke, which we did not observe with arterial hypertension or a previous myocardial infarction (Table 5).

**Table 5:** Rates of MACE and deaths according to comorbidities MACE: Major adverse cardiovascular event

Comorbidity	Rate of MACE in patients with this comorbidity	Rate of MACE in patients without this comorbidity
Renal disease	27.8% (n=10)	10.1% (n=12)
Diabetes mellitus type II	27.5% (n=11)	9.6% (n=11)
Arterial hypertension	14.2% (n=17)	14.3% (n=5)
Dyslipidemia	15.5% (n=13)	12.7% (n=9)
Previous stroke	55.6% (n=5)	11.6% (n=17)
Previous myocardial infarction	9.1% (n=1)	14.6% (n=21)
Coronary heart disease	29.4% (n=10)	9.9% (n=12)
Heart failure	33.3% (n=2)	13.4% (n=20)

We measured the serum uric acid levels of our patients at the start of the study with an average of 380µmol/l (Table 6). At the last visit after years of treatment and, most times, continuous intake of uric acid-lowering drugs, it averaged at 320µmol/l. It has to be noted that for a few participants, we could only find that the uric acid levels were in the targeted area during the follow-up, in which case we documented 359µmol/l. This means that the level should eventually be even lower at the end of the study. The number of gout attacks also significantly decreased from 3.2 to 0.3 during the last twelve months, as well as the number of tophi from 1.6 to 0.3. We did not find a relevant difference in the average blood pressure of our patients (138/83mmHg at the start vs. 136/77mmHg at the end). The rate of diabetes increased during the study from 24% (n=50) to 28% (n=44) while the renal function (measured with the eGFR) decreased from 74.9ml/min to 68.7ml/min.

**Table 6:** Comparison of parameters at the first and last measuring point of the study eGFR: Estimated glomerular filtration rate

Variable	Average at the start	Average at the end
Serum uric acid	380µmol/l	320µmol/l
Number of gout attacks during the last 12 months	3.18	0.29
Number of tophi	1.59	0.34
Blood pressure	138/83mmHg	136/77mmHg
Diabetes	24% (n=50)	28.4% (n=44)
eGFR	74.88ml/min	68.73ml/min

## Discussion

Considering all the discussed studies, allopurinol is a well-established and well-tested medication with a convenient efficacy and safety profile [2]. Febuxostat, as an alternative, presented itself as very promising in the pivotal studies APEX, FACT and CONFIRMS but also prompted questions and concerns regarding its cardiovascular safety [10]. The CARES trial (2018), which was designed for a deeper exploration of this problem [16], described barely significantly more cardiovascular deaths in the secondary endpoints in the patients taking febuxostat. Limitations of this study are the large number of participants who were lost to follow-up, partly missing assessments of co-medications and the fact that most of the excess in mortality only occurred after the discontinuation of the study drug [14,17]. The European FAST-trial (2020) reported no significant increase in cardiovascular mortality with febuxostat compared to allopurinol, and overall, almost opposite results [15]. It is important to emphasise that fewer patients presented with a previous cardiovascular disease than in the cohort of the CARES study, but an analysis of the subgroup of participants similar to the ones in CARES also did not show a significant difference. There was also a much lower rate of patients lost to follow-up in FAST [15].

From now on, observational studies coming from clinical day-to-day life are of value. We present a long-term observation of patients with confirmed gout treated with different uric acid-lowering strategies. Although not randomised, we are positive that it is still possible to conclude the clinical course.

We recruited our patients in a specialised department of a university hospital, which implies a preselection. Primary care physicians typically handle gout treatment, only referring patients to specialists under specific circumstances. Because of this, cases of gout with no complications are most likely underrepresented in our study. It may also explain that our participants were on average seven years younger than the normal gout population of Germany [18] and 87% (n=180) reported at least one comorbidity. In contrast, a case-control study from the United Kingdom documented at least one comorbidity in only 32.3% of patients with a new diagnosis of gout, even though nearly half of them developed one during the follow-up [18]. Many of our participants were taking low-dose aspirin, which shows a high cardiovascular risk. The fact that the female sex was associated with more comorbidities is frequently documented [19] and should correlate with their on average increased age. We also found higher numbers of concurrent diseases in patients initially receiving febuxostat than those receiving allopurinol or no uric acid-lowering treatment, which should explain their greater risk of suffering from a cardiovascular event in the further course of the disease. In cases of uncomplicated gout (mostly without comorbidities), there often is no need to extend the therapy beyond allopurinol as the first-line drug, so febuxostat is very less commonly found here. Compared to nationwide analyses in which febuxostat was only found in 6.5% of gout patients [18], we documented a much higher percentage of 29.3% (n=53), which may also be explained by the recruitment in a specialised department where also drugs differing from the standard medication are used. The broad spectrum of included participants is, among others, demonstrated by the blood tests taken at the start of the trial, which shows, e.g. dialysis patients with an eGFR of 9ml/min. We involved cases of newly diagnosed or never-treated gout and ones after years of strict uric acid-lowering therapy.

Although elevated liver enzymes are better known as a side effect of febuxostat [10], we documented a higher rate in the allopurinol group than in the febuxostat group during the follow-up. Even more cases were found among the patients of the other two groups, which may be the reason for frequent changes, termination or lack of start of medication. As we already established, the participants taking febuxostat most of the time were at a much higher pre-existing risk, just considering the underlying cardiovascular conditions. It remains open to discussion if the increased rate of MACE is caused by febuxostat more solely or by the comorbidities. This question unfortunately cannot be answered with this trial. Concerning the correlation of a larger number of antihypertensive and antidiabetic drugs, diuretics, low-dose aspirin, and lipid-lowering agents with documented MACE, it is inclined to accept that those patients suffer from a more severe case of the respective comorbidity. These are associated with a higher risk for MACE as frequently published [18]. The established reduction of the fractional excretion of uric acid by diuretics and low-dose aspirin [2,4,20,21] may also be a reason for this correlation. Even though lipid-lowering agents (particularly statins and fenofibrate) as well as metformin and SGLT2 inhibitors reduce the serum uric acid level [2-4,6,17], we also found more cardiovascular events in the participants taking those. This leads us to the hypothesis that the underlying medical condition as the cause of the prescription of the drug has a greater impact on the risk for MACE than the possible adverse effects of the drug itself. Higher event rates in patients with diabetes, dyslipidemia, coronary heart disease, heart failure, and a history of stroke also show this. Surprisingly, the existence of arterial hypertension did not correlate with an impaired number of MACE, which may be explained by often lacking recognition and therefore missing documentation of hypertension as an actual disease by the patients. We also did not expect the reverse association relating to a previous myocardial infarction. A possible explanatory approach might be a stricter surveillance and treatment regimen after such a severe event, as opposed to the ones for patients who only exhibit the risk factors so far. As displayed above, patients with febuxostat are taking more of the co-medications mentioned. We also found a distinct association concerning impaired renal function, which can be explained by the decreased renal clearance of uric acid in cases of renal failure [7]. The growing number of patients with diabetes across the course of the study is probably because of the chronic nature of this disease. More and more participants receive the diagnosis, but nearly nobody is cured. The decline of renal function naturally correlates with rising age, and its only minor reduction suggests a very good change of serum uric acid levels, given the common affection of the kidneys in gout patients [3].

To eliminate as many cofactors as possible to answer the issue as accurately as possible, randomised, double blind, placebo-controlled studies (RCTs) are the gold standard of research, and data from RCTs represent the highest evidence level. However, to achieve this, certain patients are explicitly excluded, which is why the studies do not represent the clinical day-to-day life. Here, it is important to examine every patient individually with all of their comorbidities and special characteristics, which often are not part of the respective RCT. This does not mean that they are not the best method to evaluate a specific benefit or side effect of a certain drug or procedure, but simply the justification for other types of trials and observations. For example, in gout therapy, a placebo group would be unethical because of well-working, already established treatments.

An obvious problem of this trial is the pre-selection of the participants, which likely results in an underrepresentation of uncomplicated cases of gout. There were also not blinding but frequent changes in medication and variable periods of therapy and observation. Some patients were already treated with uric acid-lowering drugs for many years, while others were only diagnosed with gout right before their inclusion in the trial. This can also be seen as a strength of this work because all stages of the treatment are represented. We had to collect our data from documentation which were not initially intended for this analysis, or sometimes statements of the patients by phone, which led to a certain uncertainty regarding dates or uric-acid levels. On the one hand, we have to report a drop-out of 25.5% (n=53) and unfortunately, also only a small percentage of women with gout and patients with a polyarticular manifestation. On the other hand, this study publishes data from the clinical day-to-day life. We included patients with high cardiovascular risk factors and ones with no (excluding gout as a risk factor itself). Various case scenarios are documented: years of monotherapy, adjustments of dosage, change to a different drug and also back, treatment with two different medications at the same time, intervals of no drug intake and monitoring with no therapy. In all participants, the diagnosis was secured by polarising light microscopy and the treatment was done at a specialised department of a university hospital according to the clinical guidelines. We captured all co-medications very detail, which is why we can detect and describe possible impacts. Most of the initially recruited patients were available to follow up, so our observation was possible over a long period up to 7.9 years.

## Conclusions

In the presented study, we documented a higher rate of MACEs with febuxostat as the primary uric acid-lowering drug and also showed that these patients are at a much greater risk concerning nearly all areas. Given that this constellation is probably very common, the interpretation and implementation of results from RCTs remain difficult. In particular, women and patients with non-typical cases of gout (i.e. not the typical monoarthritis affecting the MCP I) are under-represented in trials, which is why their treatment is a much greater challenge. Important are the consequent diagnosis and treatment of gout, as well as the assessment and optimisation of the commonly associated cardiovascular risk factors.
